# New U–Pb geochronology for the Central Atlantic Magmatic Province, critical reevaluation of high-precision ages and their impact on the end-Triassic extinction event

**DOI:** 10.1038/s41598-023-32534-3

**Published:** 2023-04-04

**Authors:** Alisson L. Oliveira, Mark D. Schmitz, Corey J. Wall, James L. Crowley, Antomat A. Macêdo Filho, Maria Helena B. M. Hollanda

**Affiliations:** 1 Instituto de Geociências, Universidade de São Paulo, Rua Do Lago 562, São Paulo, SP 05508-080 Brazil; 2 Department of Geosciences, Boise State University, 1910 University Drive, Boise, ID 83725 USA

**Keywords:** Environmental impact, Volcanology, Geology

## Abstract

The end-Triassic extinction (ETE) event represents one of the ‘big five’ episodes of mass extinction. The leading hypothesis for the cause of the ETE is the intrusion of voluminous magmas of the Central Atlantic Magmatic Province (CAMP) into carbon-rich sediments of two South American sedimentary basins, around 201.5 Ma. The timing of dikes and sills emplacement, however, must be considered in light of age models from CAMP rocks occurring in North America. In this work, we present new high-precision ages for critical samples in NE Brazil (201.579 ± 0.057 Ma) and Canada (201.464 ± 0.017 Ma), in order to evaluate how the South and North American magmatic events compare at the 100-ka level, and to the ETE timing. We also discuss inter-laboratory reproducibility of high-precision CAMP ages, including the ^230^Th disequilibrium corrections that are made to zircon U–Pb dates. Our findings in this newly discovered extension of the CAMP large igneous province in NE Brazil support the hypothesis that the CAMP may be responsible for the ETE through the triggering of greenhouse gas release from magma-evaporite interactions (contact metamorphism) in the South American basins.

## Introduction

The Central Atlantic Magmatic Province (CAMP)^1^ may have contributed to environmental changes that are demonstrably related to one of the ‘big five’ episodes of mass extinction^2,3^, the end-Triassic extinction event (ETE). Causality between the ETE event and the CAMP continues to be a topic of scientific debate and discussion^2–11^ within the broader context of whether and how large igneous provinces (LIPs) contribute to mass extinctions^12^.

Establishing a sequence of magmatic and biotic events for the end-Triassic is challenging, as many stratigraphic sections with important fossil and biogeochemical proxy data for the ETE are marine, while most accessible CAMP lavas and intrusions are preserved in terrestrial strata. The work of Ref.^4^ and Ref.^7^ in the western North American Cordillera and Pucara Basin of Peru presents a rare opportunity to directly date marine successions that preserve biota and a carbon isotope excursion considered to reflect the onset of the marine ETE. Direct comparisons of CAMP rocks to the terrestrial ETE have been made in the Argana Basin (Morocco), Newark Basin (USA) and Fundy Basin (Canada), where most emplaced CAMP magmas post-date the continental extinction interval^4–6,13^. The aforementioned studies have established the synchrony of the marine extinction (201.51 ± 0.15 Ma)^7^ with the age for the continental ETE (201.564 ± 0.015 Ma)^6^.

Notwithstanding this global extinction synchrony, the main hypothesis of the CAMP igneous event having a causal link to the extinction is based upon the dating of intrusions into the hydrocarbon-rich Amazonas and Solimões intracratonic basins in northern Brazil^2,3,8–10^. The continental ETE timing is derived from orbitally tuning (via Astronomic Time Scales—ATS) sediments interbedded between CAMP flows^6^ dated by the chemical abrasion isotope dilution thermal ionization mass spectrometry (CA-IDTIMS)^14^. The North Mountain Basalt (NMB) is a critical CAMP extrusive in the Fundy Basin dated by multiple works^2,4,6,10^ and, based on the ATS model of Ref.^6^, is considered to be older than the continental ETE.

Still, the precise parsing of time around the Triassic-Jurassic boundary (TJB) and the end-Triassic extinction challenges the current state-of-the-art of CA-IDTIMS U–Pb zircon geochronology at the precision necessary to resolve the relative timing of the two events and necessitates careful consideration of systematic uncertainties that are normally of minor significance. One example is the ^230^Th disequilibrium correction^15^; in general, this correction is of minor significance if the uncertainties of the data are large enough (i.e., the resulting change in the date is negligible within the measured date error), if Th is not present in large proportions in the magma to account for important variation, or if the zircons are old enough that radiogenic ingrowth overwhelms the magnitude of the daughter product disequilibrium. These are not the case for zircon CA-IDTIMS dating of Mesozoic mafic LIPs with high-precision dates (weighted mean ages around 0.03% 2 σ-uncertainties^15–17^) from single crystals that have high Th/U ratios^10^. In this vein, the analysis of independent reference materials in concert with unknown samples plays an important role in assessing the accuracy and inter-comparability of data produced via measurements in different laboratories.

**Figure 1:**
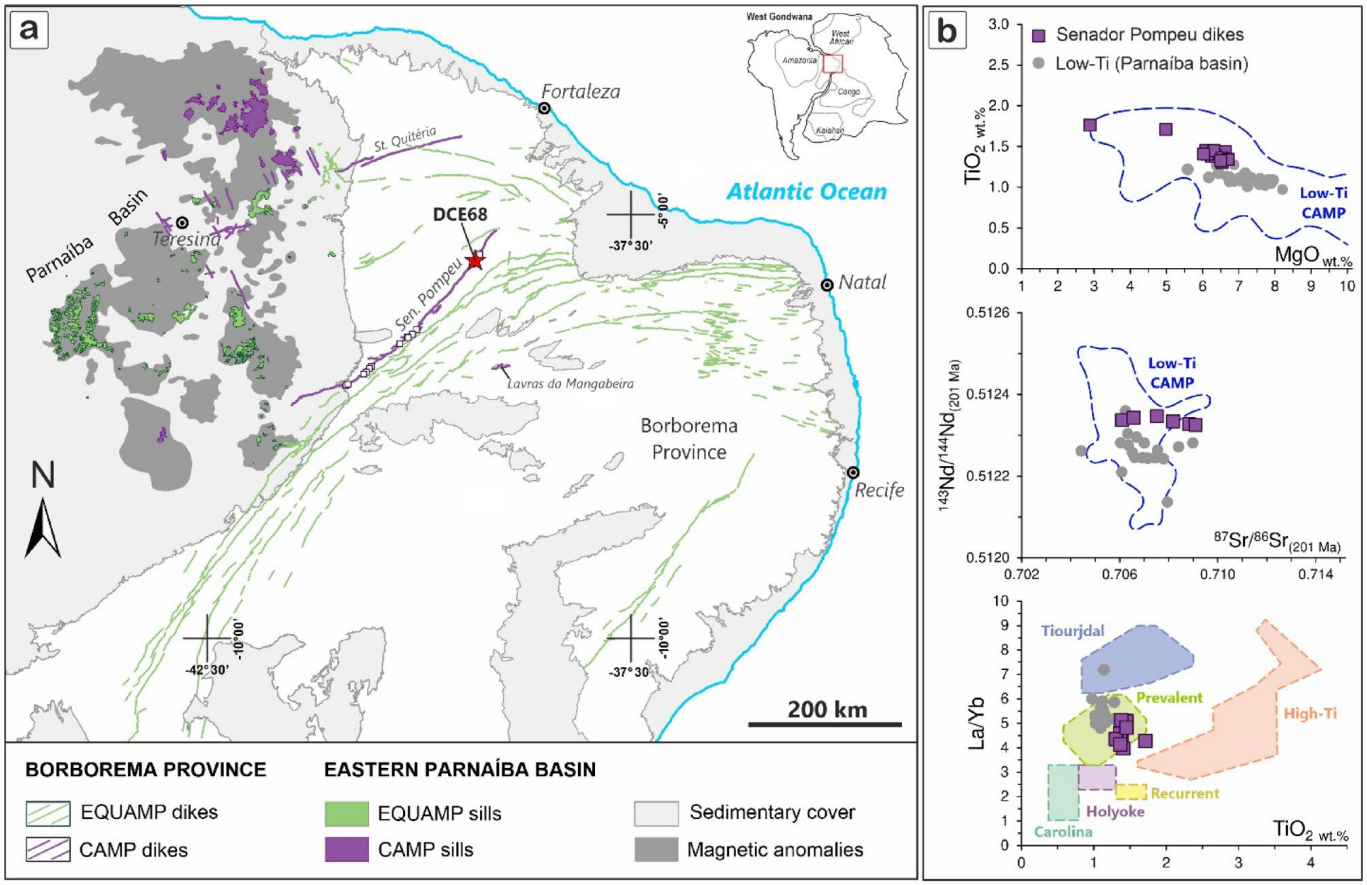
(**a**) Geological map with focus on Mesozoic LIPs in NE South America, the Equatorial Atlantic Magmatic Province^35^—EQUAMP (~ 133.3 Ma, Cretaceous) and the Central Atlantic Magmatic Province^1^—CAMP (~ 201.5 Ma Triassic) (modified from Ref.^18^). The dated sample (DCE68) of the Senador Pompeu dike swarm is highlighted by a red star. (**b**) Geochemical and isotopic aspects of Senador Pompeu dikes and CAMP Parnaíba basin sills (from Ref.^18^). Low-Ti fields and magma types from Ref.^45^.

**Figure 2:**
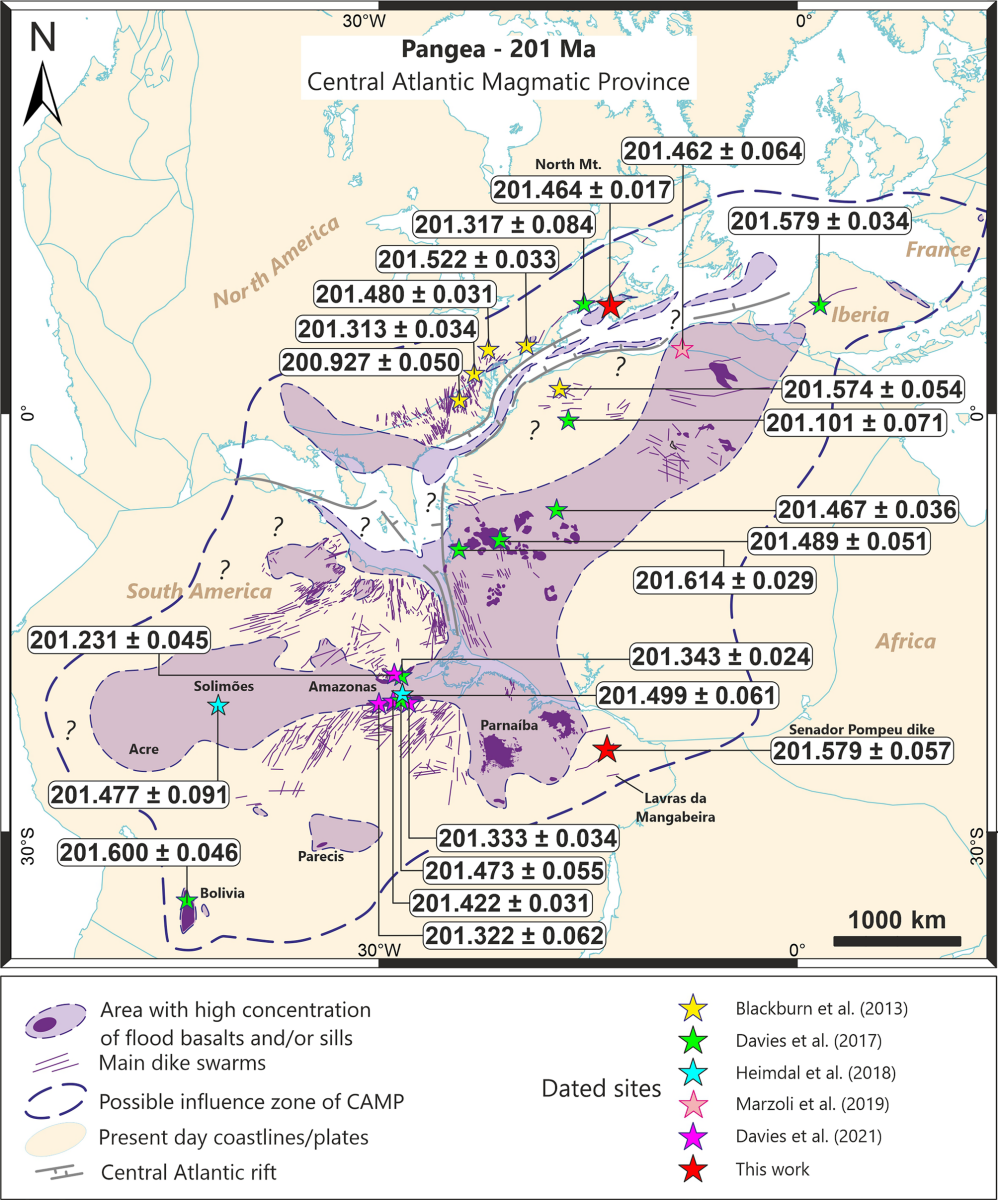
Reconstruction map of the Central Atlantic area during the Triassic-Jurassic Boundary (based on combined models of Ref.^65^). Position of sills, flows and dikes are shown with locations of critical samples dated by the CA-ID TIMS method (2 σ uncertainty). New ages for the North Mountain Basalt (Canada) and the Senador Pompeu dike (NE Brazil) are presented. Previously dated samples^2,3,6,10^ were recalculated to new ^230^Th disequilibrium corrections. Map created using software GPlates v2.3 (https://www.gplates.org/) and ArcGis Software Package version 10.3.1 (http://desktop.arcgis.com/en/arcmap/).

In this work, we further test the CAMP-ETE causality hypothesis by presenting a high-precision CA-IDTIMS U–Pb zircon age for a previously undated CAMP dike swarm in NE-Brazil^18^. The Senador Pompeu dike is representative of a ~ 360 km long dike swarm hosted in the Precambrian basement of the Borborema Province, located ~ 1500 km eastward of the Amazonas Basin and exhibiting similar geochemical and isotopic characteristics to low-Ti magmas of the CAMP^18–20^. Overall, the Senador Pompeu dikes (Fig. 1) differs from other neighboring Equatorial Atlantic Magmatic Province (EQUAMP) dikes by having higher MgO (> 6 wt.%), lower contents of incompatible trace elements (Sr < 250 ppm), more radiogenic Nd isotope signatures (eNd > -1.5) and higher Ti/Zr ratios^18^. Within the Borborema Province, two dikes have been interpreted as part of the CAMP by geochemical and geochronological proxies, the Senador Pompeu and the Santa Quitéria dikes^18^. These two dike occurrences have a strong correlation to smaller dikes and two sills recently charted in the southeastern and northeastern border of the Parnaíba basin^18,19,21^. The largest proportion (*ca* 295 km) is hosted in the Precambrian basement, while ca. 65 km is underneath Paleozoic sedimentary units in the basin^18,22^ (Fig. S1-A). This new dike swarm, together with the Lavras da Mangabeira basalts^23^, represents the easternmost occurrence of the CAMP magmatism in South America (Fig. 2), extending the province outcropping border approximately 800 km further east in South America. We present this result together with new high-precision dates of the North Mountain Basalt (NMB) and discuss the state of high-precision geochronology for the CAMP and a re-evaluation of its relation to the ETE, particularly in light of consonant ^230^Th disequilibrium corrections for the database of extant U–Pb zircon ages. Moreover, we used the 100 Ma EARTHTIME synthetic U/Pb solution (ET100)^24^ to demonstrate that the accuracy of our dating experiments is in accordance with other laboratories worldwide^25^. For this study, both the ET100 and the NMB sample serve in the role of reference samples for comparison with other published data around the TJB and ETE.

## Methodology

Our North Mountain Basalt zircon crystals are derived from the same sample (NMB03) dated by Ref.^4^, Ref.^6^ and Ref.^2,10^. The zircon separation for NMB03 followed a conventional procedure of rock crushing, milling, magnetic and density concentrations, from which reasonably large crystals (> 150 µm) were selected for dating. The Senador Pompeu dike sample (DCE68) followed the chemical dissolution method of Ref.^26^ for zircon concentration that allowed a recovery of small crystals (< 80 µm) that were not recoverable by conventional separation procedures.

Single crystal U–Pb zircon CA-IDTIMS analyses were done at the Isotope Geology Laboratory at Boise State University, following a modification of the chemical abrasion method of Ref.^14^. Annealing of NMB03 crystals was done at 900 °C for 60 h in a muffle furnace; the DCE68 sample was annealed under the same conditions during the chemical dissolution separation. Zircons were then chemically abraded in individual 300 µL Teflon PFA microcapsules in 120 µL of 29 M HF with a trace of dilute HNO_3_ in a single aggressive step for 12 h at 180 or 190 °C. After chemical abrasion, the residual grains were rinsed, reloaded into microcapsules, and spiked with the EARTHTIME^27,28^ mixed ET2535 tracer solution. The spiked grains were then totally dissolved (29 M HF at 220 °C for 48 h) and re-equilibrated (6 M HCl at 180 °C for 12 h) in Parr vessels, after which U and Pb were separated using an anion-exchange chromatographic procedure modified from Ref.^29^, eluted together and dried with 2 µL of 0.05 N H_3_PO_4_. Pb and U were loaded on a single outgassed Re filament in 2 µL of a silica-gel/phosphoric acid mixture^30^. U and Pb isotopic measurements were made on Isotopx Phoenix or Isoprobe-T multicollector thermal ionization mass spectrometers equipped with an ion-counting Daly detector for single collector analysis of Pb isotopes, and Faraday cups with 10^12^-Ω resistors for static multicollection of uranium isotopes. U and Pb mass fractionation were corrected using the known ratios (^202^Pb/^205^Pb = 0.999239 ± 0.0265% 1 σ), ^233^U/^235^U = 0.995062 ± 0.0054% 1 σ) of the ET2535 tracer solution. All common Pb in the zircon analyses was attributed to laboratory blank based upon numerous total procedural blank measurements. Although there is some intrinsic ^204^Pb within the ET100 solution we follow other published literature in assigning all apparent common Pb to laboratory blank. Experiments show that this assumption does not introduce significant bias in the calculated apparent ^206^Pb/^238^U date of the solution. ^206^Pb/^238^U ratios and dates for zircon analyses were corrected for initial ^230^Th disequilibrium using a Th/U_[magma]_ based upon measured whole rock host basalt compositions. No ^230^Th correction was applied to ET100 solution analyses.

U–Pb dates and uncertainties for each analysis were calculated using the algorithms of Ref.^31^ with a ^235^U/^205^Pb ratio of 100.233 ± 0.05% (1 σ). All age calculations are based on the decay constants of Ref.^32^ and the terrestrial ^235^U/^238^U of Ref.^33^. The quoted age error includes analytical uncertainties of counting statistics, spike subtraction and Pbc correction, and is appropriate in comparisons with other ^206^Pb/^238^U ages obtained with the EARTHTIME spike. If used in comparison with ages derived from other U–Pb methods or decay schemes (e.g., ^40^Ar/^39^Ar), the uncertainty in the spike U/Pb ratio and the ^238^U decay constant must be considered. Therefore, the errors for individual analyses are in the form of ± X [Y/Z], where X is analytical uncertainty, Y is the tracer uncertainty (used for comparison with other U–Pb methods), and Z is the combined analytical, tracer, and ^238^U decay constant uncertainty (i.e., 0.106%). Y and Z must be added to X in quadrature. All errors are reported as 2 σ; the probability of fit of sample variance to a normal distribution expected from analytical errors was assessed using the reduced chi-squared, or mean squared weighted deviations (MSWD) statistic^34^.

## Results

### ET100

**Figure 3:**
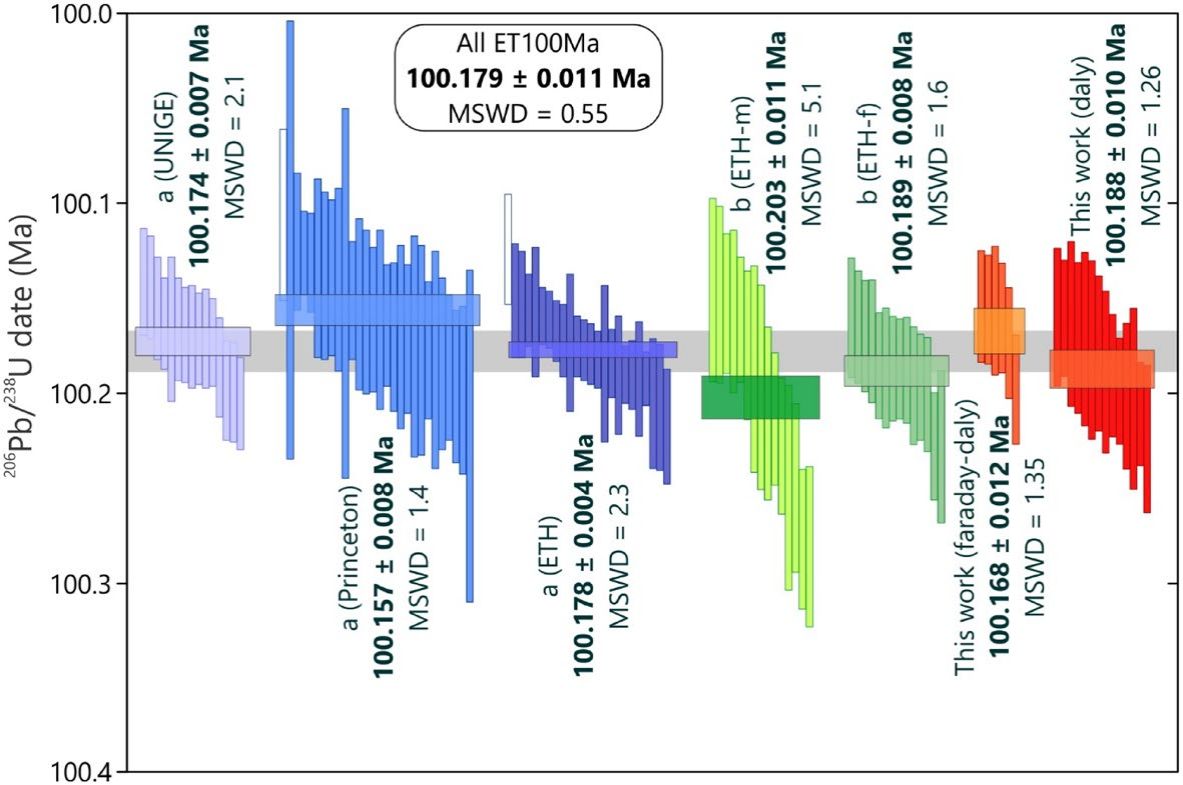
Compilation of ET100Ma solution ^206^Pb/^238^U apparent ages using the ET2535 spike (from a—Ref.^25^, b—Ref.^38^ and this work). Blank boxes represent outliers not included in weighted mean age calculations. Different laboratories are discriminated in parenthesis, as well as Pb analyses measured by electron multipliers (m), Faraday cups (f), Daly photomultiplier (d) or a combination of methods (f-d). The grey bar through the middle of the plot is centered on 100.179 ± 0.011 Ma, which illustrates a ± 0.011% variation between laboratories.

Twenty aliquots of the ET100 solution^24^ were prepared with a range of radiogenic ^206^Pb (Pb*) contents from 1118.1 to 6.5 pg. All aliquots were equilibrated with the ET2535 tracer and purified by anion exchange chromatography following the same protocol used for zircon analysis. Analyses of > 800 pg of Pb* were measured by multicollector Faraday–Daly measurements, where the Faraday-Daly gain was controlled by analyzing the ^205^Pb isotope on both collectors. All other ET100 analyses (i.e., < 100 pg of Pb*) followed the same analytical protocol used for unknown zircons (i.e., using the Daly detector for Pb isotopes and Faraday cups for U isotopes). A weighted mean ^206^Pb/^238^U date of 100.168 ± 0.012 [0.032/0.112] Ma (n = 6, MSWD = 1.35) was calculated from the large (i.e., > 800 pg) Pb* aliquots. When considering only the solutions with less than 100 pg of Pb*, which are more comparable in Pb* and analytical protocol for the CAMP zircons, a ^206^Pb/^238^U date of 100.188 ± 0.010 [0.031/0.112] Ma (n = 14, MSWD = 1.26) was obtained (Supplementary File Table 1, Fig. 3). The two results cannot be distinguished at the 95% confidence interval, and provide a first approximation of the ~ 0.01% limits of analytical resolution using our U–Pb isotope dilution techniques.

### North Mountain Basalt

**Figure 4:**
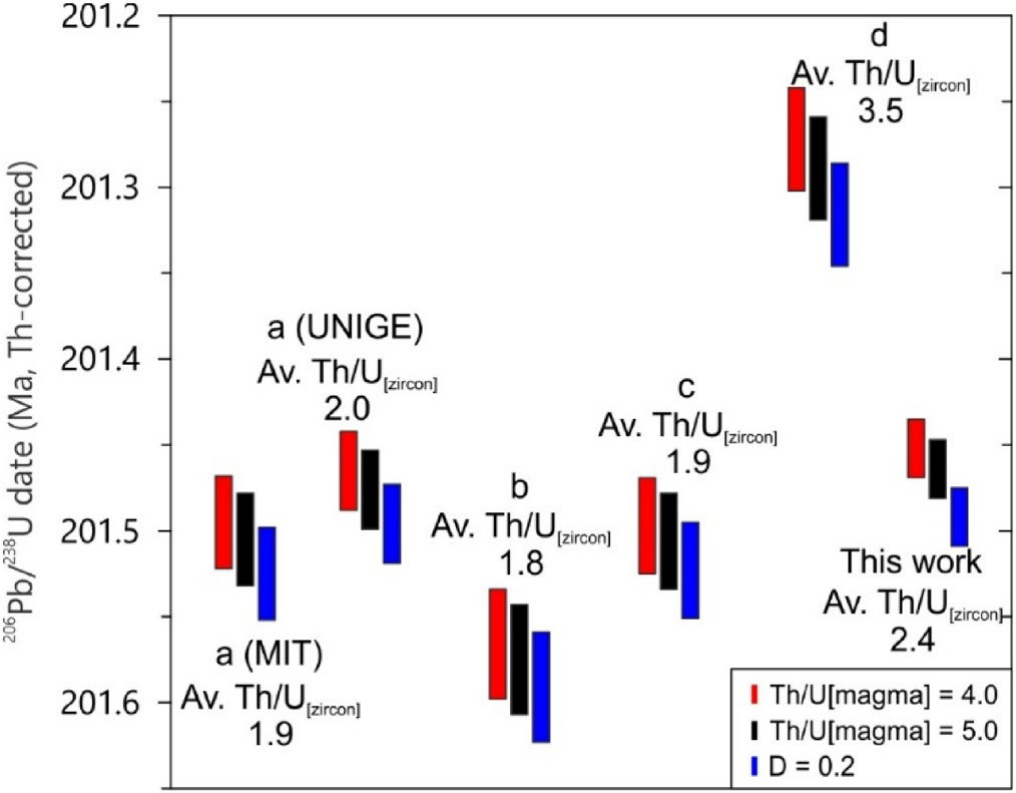
^206^Pb/^238^U weighted mean ages for the NMB03 sample using different Th/U_[magma]_ correction values. (a)—Ref.^4^, (b)—Ref.^6^; (c)—Ref.^2^; (d)—Ref.^10^ and this work. Note that depending on the Th/U_[zircon]_ value (expressed as average) and the weighted mean age error, a different correction may imply in discordant ages for the same analysis. Ages from Ref.^4^ were also recalculated from previous EARTHTIME tracer calibration based on the corrections made by Ref.^7^.

**Figure 5:**
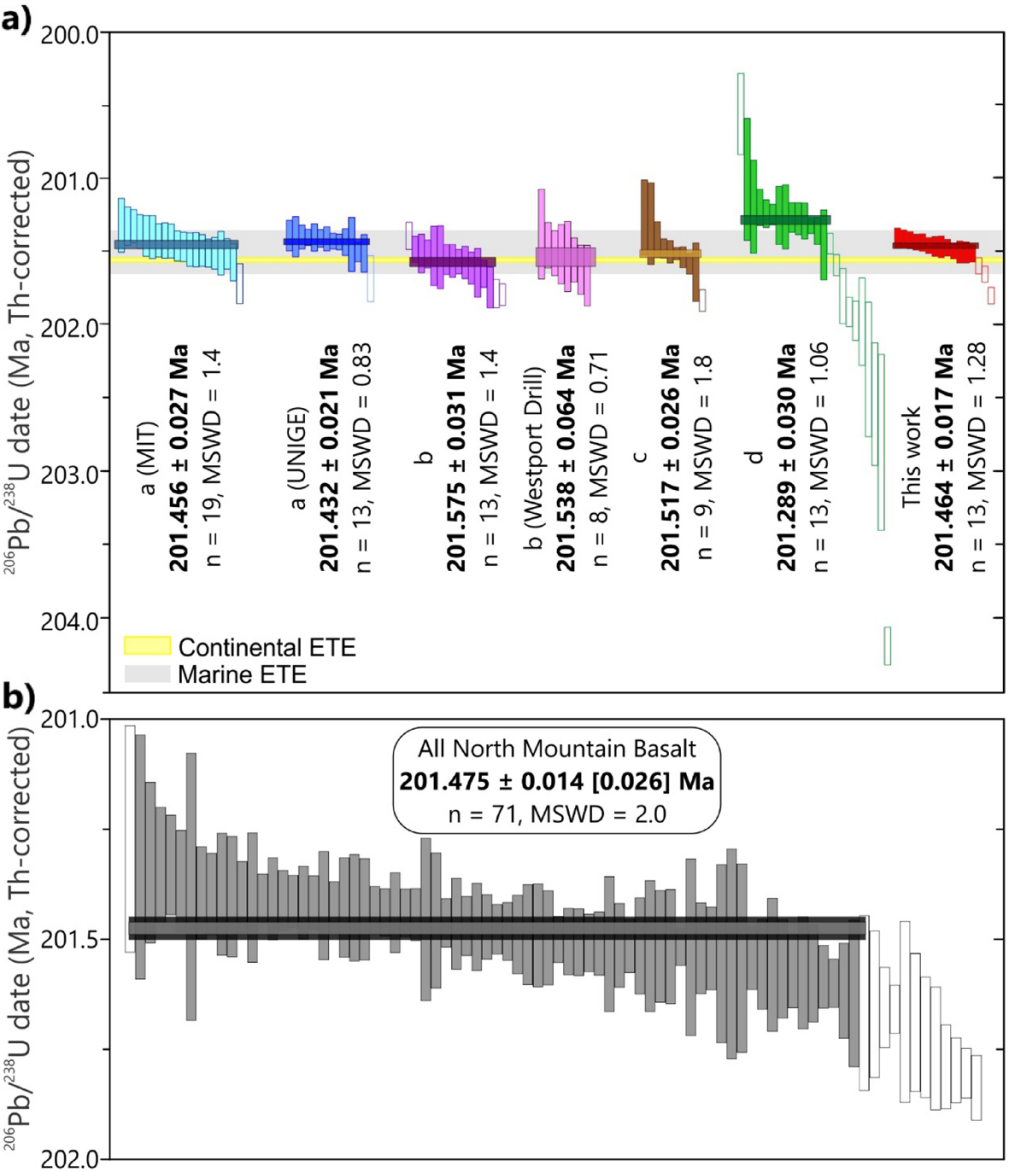
(**a**) Comparison of ^230^Th disequilibrium-corrected ages for the North Mountain Basalt (NMB03 + Westport Drill core). All original data published by (a)—Ref.^4^, (b)—Ref.^6^; (c)—Ref.^2^; (d)—Ref.^10^ and this work were corrected using a common Th/U magma ratio of 5.0 ± 0.5 (based on values from Ref.^49^. Ages from Ref.^4^ were recalculated from previous EARTHTIME tracer calibration based on the corrections made by Ref.^7^. Blank boxes represent outlier crystals not included in weighted mean age calculations. Continental and marine ETE timing are from Ref.^6^ and Ref.^7^, respectively. (**b**) Compilation of all analyses for a best estimate weighted mean age for the North Mountain Basalt, outlier crystals were removed by failing a modified version of the Thompson’s Tau rejection test. The uncertainty is shown as the weighted mean standard error, and also the inflated in quadrature by 0.011% dispersion from the ET100 U–Pb solution threshold in brackets.

Sixteen single crystals from the NMB03 sample were analyzed (Supplementary File Table 1). We used two different mass spectrometers and two different chemical abrasion temperatures to compare different dating protocols. From the 16 analyses, 9 were done by abrasion at 180 °C from which 8 were analyzed on the Isoprobe-T and 1 on the Phoenix spectrometer; the other 7 analyses were treated at 190 °C, where 3 were analyzed on the Isoprobe-T and 4 on the Phoenix spectrometer. The differing chemical abrasion temperatures and mass spectrometers were used to assess Pb loss, dead-time corrections, and reproducibility of data. The experiments, however, did not render systematically different results. Of the 16 individual grains analyzed, 13 yielded concordant and equivalent isotope ratios from which we calculated a weighted mean date of 201.464 ± 0.017 Ma [0.1/0.24] Ma (MSWD = 1.28; Figs. 4, 5). Another 3 analyses were slightly but resolvably older, compatible with previous reports^2,6,10^.

### Senador Pompeu dike

**Figure 6:**
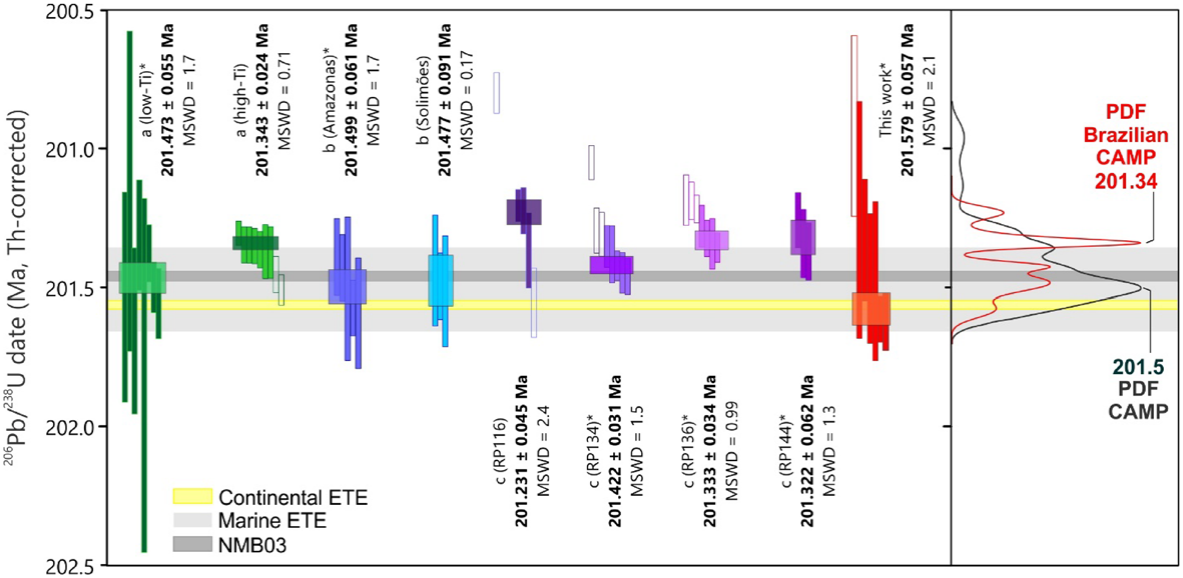
Revised ages for South American CAMP rocks corrected to the same Th/U value of 5.0 ± 0.5. (a)—Ref.^2^; (b)—Ref.^3^; (c)—Ref.^10^ and this work. Continental and marine ETE timing are from Ref.^6^ and Ref.^7^, respectively. Age for the NMB03 sample is the same shown in Fig. 5b. Asterisk (*) indicates samples from low-Ti (< 2%wt. TiO_2_) magma types, presumed as causers of the end-Triassic extinction (ETE) event. PDF (probability density functions) curves are derived from U–Pb ages, black is relative to the entire CAMP magmatism (minus South American samples), red is derived from South American CAMP dates shown in this figure.

Sample DCE68, a low-Ti tholeiite from the Senador Pompeu dike (Supplementary file) was initially presumed to be part of the *ca* 133 Ma EQUAMP^35^ due to its outcropping proximity. However, geochemical, and isotopic signatures revealed that CAMP rocks were intermingled in the EQUAMP area^18–20^. A total of seven single crystals were analyzed, where one was considerably younger and treated as biased by Pb* loss. A weighted mean age of 201.579 ± 0.057 [0.083/0.23] Ma (MSWD = 2.1) was calculated from the six concordant crystals (Supplementary File Table 1, Fig. 6).

## Discussion

### Assessment of inter-laboratory U–Pb isotope ratio reproducibility

The synthetic ET100 U–Pb solution is an effective reference standard for assessing the reproducibility of both sample-spike equilibration and mass spectrometric techniques in isotope dilution thermal ionization mass spectrometry^25^. The results presented by Ref.^25^ for three different laboratories (100.173 ± 0.003 Ma) exemplify how the ET100 solution is a generally optimal reference material to check the repeatability and accuracy of a dating experiment, however, it also highlights the necessity of highly controlled analytical conditions. We applied the same sample preparation protocols to the ET100 solution as used in U–Pb isolation from zircon, including high temperature and pressure heating, multiple phase transformations (chlorides and fluorides), and ion chromatographic separation in order to promote sample-spike equilibration and ionization behavior in the mass spectrometer source comparable to U–Pb zircon analyses.

The apparent age of 100.179 ± 0.011 Ma (MSWD = 0.55) for the ET100 solution obtained here (Fig. 3) agrees within errors with other published ages for this solution that used the ET535 spike (100.202 ± 0.018 Ma^36^; 100.209 ± 0.038 Ma^37^), and with a more precise and accurate dating experiment (100.189 ± 0.008 Ma^38^) that used the ET2535 spike. A detailed compilation of results for the ET100 solution from Ref.^25^, Ref.^38^ and this work (Fig. 4) quantifies the variance of results between laboratories, which can be calculated considering the experiments’ weighted means and standard errors or their full probability density functions. The latter are irresolvable for the seven experiments illustrated in Fig. 4, while the former technique does exhibit excess variance over analytical uncertainties, as assessed via the reduced chi-squared statistic, or mean squared weighted deviation (MSWD = 9.3) for this compilation. When this excess variance is accommodated by multiplying the ensemble standard error by the square root of the MSWD, then both strategies arrive at a similar metric of external reproducibility between ET100 solution experiments (and thus inter-laboratory reproducibility) of 0.011% at the 95% confidence interval.

### Intercalibration of high-precision ages for the CAMP

The ^230^Th-disequilbrium or ‘Th/U correction’ is a necessary step in precise and accurate ^238^U–^206^Pb geochronology of Phanerozoic zircons. For the end-Triassic samples under consideration, the Th/U correction is of similar magnitude to the calculated standard error of their ages. The Th/U correction is computed via the ratio of the mineral/melt partition coefficients (D) for Th and U^39,40^. Theoretically, if the partition coefficient ratio (D_Th/U_) is invariant then the correction is of constant magnitude and systematic uncertainty. Such is not the case for natural magmatic systems, however, because D_Th_ and D_U_ vary depending on fO_2_, melt composition, H_2_O saturation and more^41–43^. If partition coefficients are not invariant, then Th/U corrections are made using the measured Th/U of the zircon itself and an assumption for the Th/U of the liquid from which the zircon crystallized (Th/U_[magma]_), which together proxy the effective mineral/melt partition coefficient. The Th/U_[magma]_ is generally assumed to be constant during zircon crystallization.

Most studies of CAMP zircon geochronology have applied the constant Th/U_[magma]_ assumption for ^230^Th-disequilbrium based upon observations and modeling of Th/U variations in magmas^6^. However, we note that some recent studies have used a constant experimental D_Th/U_ between zircon and magma of 0.2^2,10^. For comparison, calculating a Th/U_[magma]_ ratio from the Th/U_[zircon]_ measured during CA-IDTIMS analyses with a D_Th/U_ value of 0.2 results in an average Th/U_[magma]_ value of 17.4^10^ for the hypothetical CAMP magmatic liquids. This ratio is much higher than the Th/U ratio of most silicate magmas (2–6)^15^, including the range of Th/U from 2.0 to 6.8 recorded in over 500 samples of CAMP lavas, diabases, and their differentiates that range from < 50 to > 70 wt.% SiO_2_^44–46^. This inconsistency suggests that the zircon D_Th/U_ for more evolved granitic liquids is not applicable to the evolution of mafic systems—a conclusion also reached for mid-ocean ridge gabbros^47,48^. A similar notion was also made by Ref.^6^, that stated that Th/U_[magma]_ compositions of 4.0 should be viewed as minimum values, but ratios much higher than 6.5 are unlikely to occur. Experimental studies have documented a strong dependence of D_U_ on fO_2_ and corresponding variations in D_Th/U_^42^, which may be particularly relevant to mafic intrusive magmatic differentiates^47^. We conclude from the current state of experimental and observational petrology that the constant D_Th/U_ assumption is likely to produce more systematic bias in the ^230^Th-disequilbrium correction than the constant Th/U_[magma]_ assumption in mafic systems.

The Th/U_[magma]_ ratio used for low-Ti CAMP rocks in most geochronological studies has been around 4.0^3,4,6^. We calculated a Th/U_[magma]_ ratio of 5.03 ± 0.50 (n = 11) from whole-rock analyses three different flow sequences characterized at the Digby area (Nova Scotia) as indicative of this ratio for the NMB^49^. This Th/U is comparable to the aforementioned broader literature for CAMP intrusives. Using a Th/U_[magma]_ value of 5.0 ± 0.5 for our NMB03 analysis, the Th/U-corrected weighted mean date is 201.464 ± 0.017 Ma. However, when using a Th/U_[magma]_ ratio of 4.0 ± 0.5 or a D_[zircon-magma]_ value of 0.2 (av. Th/U_[magma]_ = 11.1), the calculated dates change to 201.452 ± 0.018 Ma and 201.492 ± 0.017 Ma, respectively. Similar results are also seen for other NMB03 dating experiments (Fig. 4). Consequently, comparing ages calculated from different Th/U corrections might produce apparent dispersion that could be misinterpreted as of geological significance at the level of temporal resolution sought for the ETE (i.e., tens of ka). For this reason, we harmonized the Th/U correction for all published NMB and Brazilian CAMP zircons, using the equations of Ref.^39^ and Ref.^40^, where the various ^206^Pb/^238^U ratios and Th/U_[zircon]_ and Th/U_[magma]_ ratios are obtained from published values in the original paper (when applicable), and the λ238 and λ230 values are based on the decay constants of Ref.^32^ and Ref.^50^. We used the equation in supplementary Table 2 to recalculate the Th/U corrections of every published single crystal date to a common Th/U_[magma]_ ratio of 5.0 ± 0.5 (based on values from Ref.^49^).

The resulting NMB03 weighted mean ^206^Pb/^238^U ages from Ref.^6^, Ref.^2,10^, Ref.^4^ and this work are not all concordant (Fig. 5) despite being derived from the same sample. Single crystal dates range from 200.568 ± 0.276 Ma to 204.187 ± 0.129 Ma and the calculated weighted mean ages vary from 201.289 ± 0.030 Ma to 201.575 ± 0.031 Ma. As all of these measurements utilize a common EARTHTIME spike calibration, and are derived from the same rock volume, we have aggregated the analyses into a single distribution for the purpose of deriving an improved estimate of the eruption and crystallization of the North Mountain Basalt. The data of Ref.^10^ contains many crystals tailing to younger dates typical of unmitigated Pb loss; for this reason, we removed this data set from further consideration. Slightly to significantly older xenocrysts are reported in all studies of the NMB and were discussed by Ref.^10^ within the context of predicted zircon saturation in the basaltic magma. We used the modified Thompson Tau test^51^ to objectively remove a single younger outlier, and the tail of older outliers, to arrive at an updated crystallization and eruption age for the NMB of 201.475 ± 0.014 Ma (MSWD = 2.0, n = 71, 95% confidence interval; Fig. 5). When the systematic interlaboratory variance of 0.011% from the aforementioned ET100 solution experiments is considered, the resulting NMB age is 201.475 ± 0.026 Ma (Fig. 5). Adding this systematic variance to other dates (such as CAMP dates) could also imply in larger errors that might incur closely unconcordant dates to overlap, which can have potentially significant consequences.

The selection of crystals to include in weighted mean age calculation is also critical^52^. If LIPs zircons crystalizing from mafic melts are formed from evolved fractionated liquids^10^ or elevated oxygen fugacity melts^47^, the derived dates should reflect late-stage crystallization close to the emplacement time of the igneous body. Ref.^10^ propose that the older crystals represent time of emplacement more accurately in the mafic LIPs setting, since the extremely high U (and Th) contents found on these zircons incur severe radiation damage and likely Pb loss after crystallization. Apparently younger crystals thus may contain a residual bias from unleached U-rich domains even after chemical abrasion. We followed the proposed model of Ref.^10^ and calculate a weighted mean age from the oldest zircons, considering the one younger crystal as an outlier; this approach was used for all South American samples (Fig. 6).

### End-Triassic extinction event and South American CAMP ages reconsidered

The ETE event is marked by at least three carbon isotope excursions (CIE)^8^. These CIEs were hypothesized to be the product of intense volatile degassing from contact metamorphism of sills intruding carbon-rich sediments and evaporites in the Amazonas and Solimões intracratonic basins^3,9^. A total gas buildup of *ca* 88,000 Gt CO_2_ has been estimated, and the volume of gases released into the atmosphere could account for most of the observed carbon anomalies around the TJB. Mantle-derived carbon is considered unlikely to cause the observed CIEs, while extremely depleted biogenic CH_4_ is not needed to replicate the modeled necessary thermogenic degassing^8^. Even so, the contribution of volcanic toxic gases and thermogenic carbon released from sediments-sills interactions are the likely causes of the catastrophic environmental perturbation that ultimately led to the mass extinction event observed at the end of the Triassic Period^3,8,9,53–55^.

The Parnaíba Basin presents a similar feature to the other two Brazilian basins, where mafic sills can extend continually for 200 km, have an estimated maximum thickness of *ca* 500 m^56,57^, and intrude the Canindé (fine sandstone, siltites and black shales), Serra Grande (sandstone and shales), and Balsas (clastic-evaporitic complex) groups^57^. The Pimenteiras Formation (Canindé Group) has more than 500 m in thickness and is composed of dark shales rich in organic matter^58,59^, where total organic carbon reaches up to 16.60 wt.%, while rock–eval pyrolysis was measured to a high of 51.43 mm HC/g^59^. Additionally, vitrinite reflectance values show an increase in T max (451 °C) and the presence of talc, chlorite and illite can be associated with the thermal influence of igneous intrusions^58,59^. Overall, there is a direct correlation between hydrocarbon generation and magmatic events in the Parnaíba Basin^56,58–60^.

In models calling upon South American CAMP magmatism as the main source of paleoenvironment disturbance in the end-Triassic crisis, the ETE must be synchronous or younger than the CAMP intrusions into those sedimentary strata. The magma types of the CAMP are often grouped as low- (< 2%wt. TiO_2_) or high-Ti (> 2%wt. TiO_2_), and overall, it remains evident that the low-Ti type is synchronous to the high-Ti type^10^, but the slightly older pulse is currently only represented by low-Ti rocks (Fig. 6). The Senador Pompeu dike from the CAMP in NE Brazil represents one of the easternmost occurrences of this LIP in South America and is analogous to the low-Ti ‘Prevalent’ CAMP type (petrological characteristics available are as Supplementary File) widespread in the Amazonas and Solimões basins^3,18^. Recent ages published by Ref.^2,10^ and Ref.^3^ (with recalculated Th/U corrections, Fig. 6, Supplementary File Table 2) indicate emplacement between 201.499 ± 0.061 to 201.231 ± 0.045 Ma for the Brazilian CAMP magmatism, generally younger than the continental ETE (201.564 ± 0.015 Ma^6^). In contrast, our Senador Pompeu dike age of 201.579 ± 0.057 Ma is slightly older than other dated Brazilian CAMP intrusions in the Solimões and Amazonas basins (Figs. 2 and 6), while also being coeval with the marine (201.51 ± 0.15 Ma^7^) and terrestrial ETE timing. This new dated CAMP occurrence, together with the Tarabuco sill in Bolivia, represents the first episodes of the CAMP magmatism in South America (see the probability density functions in Fig. 6).

Several dike swarms are mapped in the surrounding basement of the Parnaíba Basin (Fig. 1), but when those dikes reached pre-existing sedimentary layers, they were apparently arrested to form layered intrusions, since rare dikes^61^ and abundant sills^57,62^ occur in the Parnaíba Basin. Therefore, dike swarms (here, chronologically represented by the Senador Pompeu dike) likely represents a complex plumbing system that fed sills in the Parnaíba Basin, similar to other Paleozoic intracratonic basins of South America^3,60,63^. Thus, the recent finding of the Senador Pompeu dike suggests that there may be additional as yet undated early low-Ti magmatism in the South American sedimentary basins. The samples dated by Ref.^3^ at the Amazonas and Solimões basins have single crystal dates that are synchronous to the continental ETE timing, but the overall weighted mean age (at the 2 σ level) just barely position them at the same age interval as the extinction. If the CAMP magmatism is indeed responsible for the major climatic changes in the TJB, it seems evident that the first magmatic pulse is the only episode that predates the current extinction timing, which is similar to other LIP-related mass extinction driving mechanisms^64^, and a conclusion also reached by Ref.^2^.

Even so, almost all North American samples^6^ post-date the CIE and extinction intervals. The NMB, which was considered slightly older than the ETE^6^, now post-dates it by almost 80 ka, while most dated samples in the Amazonas Basin^2^ are similarly younger than the ETE. Nonetheless, the earliest CAMP intrusions, including our new Senador Pompeu dike and the Tarabuco sill in South America, the Kakoulima intrusion in Guinea, the Argana sill in Morocco, and the Messejena dike in Iberia, all located at the eastern border of the province are synchronous with the ETE at a resolution of ≤ 100 ka. This first pulse, however, is not the major magmatic emplacement episode of the province, shown by the number of dated rocks in the CAMP province that indicates a more prominent volume on the second peak, around 201.52 Ma (Fig. 6). Therefore, degassing of sediments in Brazilian intracratonic basins, such as the Parnaíba, Amazon and Solimões^2,3,8,9,56,59^, could have all joined forces to play a role in the End-Triassic crisis, but it remains necessary to establish the widespread occurrence of the first CAMP magmatic pulse that seems to account for most of the greenhouse gases released around the ETE and TJB.

## Conclusions

We report a precise age of a newly discovered low-Ti CAMP dike swarm in NE Brazil and present a recalculation of all high-precision U–Pb zircon CAMP ages using a single Th/U correction scheme based on a mafic magma composition. The 201.579 ± 0.057 Ma Senador Pompeu dike in NE Brazil represents one of the oldest occurrences of the CAMP in South America, and its synchrony with the ETE supports the hypothesis that intrusions into the hydrocarbon-rich sedimentary Brazilian basins may have been the trigger for climatic and biotic upheaval around the Triassic-Jurassic boundary, even though this magmatic pulse is still underrepresented in the current Brazilian CAMP database. Moreover, we show how a controlled interlaboratory analytical routine and ^230^Th disequilibrium corrections can impact high resolution interpretations and should be considered thoroughly when applying the CA-IDTIMS method to derive results at the < 0.03% 2 σ uncertainty level.

## Supplementary Information

Supplementary Information 1.

Supplementary Information 2.

Supplementary Information 3.

## Data Availability

All data generated or analysed during this study are included in this published article (and its Supplementary Information files).
