# Supplementary material for: New U–Pb geochronology for the Central Atlantic Magmatic Province, critical reevaluation of high-precision ages and their impact on the end-Triassic extinction event
Source: Sci Rep. 2023 Apr 4;13:5485. doi: 10.1038/s41598-023-32534-3 (PMC10073112; doi:10.1038/s41598-023-32534-3)
Supplement: Supplementary file 3 — Supplementary Information 3. [file 41598_2023_32534_MOESM3_ESM.docx]

Supplementary file - New U-Pb geochronology for the Central Atlantic Magmatic Province, critical reevaluation of high-precision ages and their impact on the end-Triassic extinction event

The Senador Pompeu dikes, NE South America

There are two large igneous provinces (LIPs) in NE Brazil (Figure 1-a, main text): the Equatorial Atlantic Magmatic Province (EQUAMP)^1,2^, and the Central Atlantic Magmatic Province (CAMP)^3,4^, dated within the Cretaceous (~133.3 Ma) and Triassic (~201.5 Ma) Periods, respectively. Both LIPs crop out within the Parnaíba intracratonic basin and its adjacent basement rocks, the Paleoproterozoic Borborema Province. Remarkable advances in recent years have improved the geological cartography of these events^1,2,5–10^, and the provinciality of sills, dikes and flows have been especially constrained based on geochemistry, isotopes, paleomagnetism and geochronological data^2,3,9–15^.

The Senador Pompeu swarm is an N45E-oriented structure with a total length of ca. 360 km, where individual bodies reach *ca* 50 m in width. Magnetic anomalies indicate that the dikes extend from the central region of the Borborema Province to the SE flank of the Parnaíba basin^2,7^. They are holocrystalline/subophitic diabases (Fig. S1-B) composed of plagioclase (labradorite-bytownite), augite and magnetite, with the subordinate occurrence of pigeonite, apatite, sulfides, spinels, and zircon (Fig. S1-C). Geochemically, it is a low-Ti (TiO_2_ <2 wt.%) correlated to the Prevalent CAMP type (Figure 1-b, main text), with moderate crustal contamination/alteration evidenced by slightly ^87^Sr/^86^Sr enrichment and unvarying radiogenic ^143^Nd/^144^Nd compositions^2^. Trace elements show enrichment in incompatible elements (Ba, Sr, Cu and Ni range from 568-158 ppm, 245-186 ppm, 227-76.4 and 60-11.3, respectively), while rare earth elements yield La/Yb ratios around 3.0-2.7 and a negligible Eu anomaly.

Additional information

Supplementary File Table 1 – U-Th-Pb isotopic data

Supplementary File Table 2 – ^230^Th disequilibrium corrections for published U-Th-Pb CA-IDTIMS Central Atlantic Magmatic Province zircon ages.

| 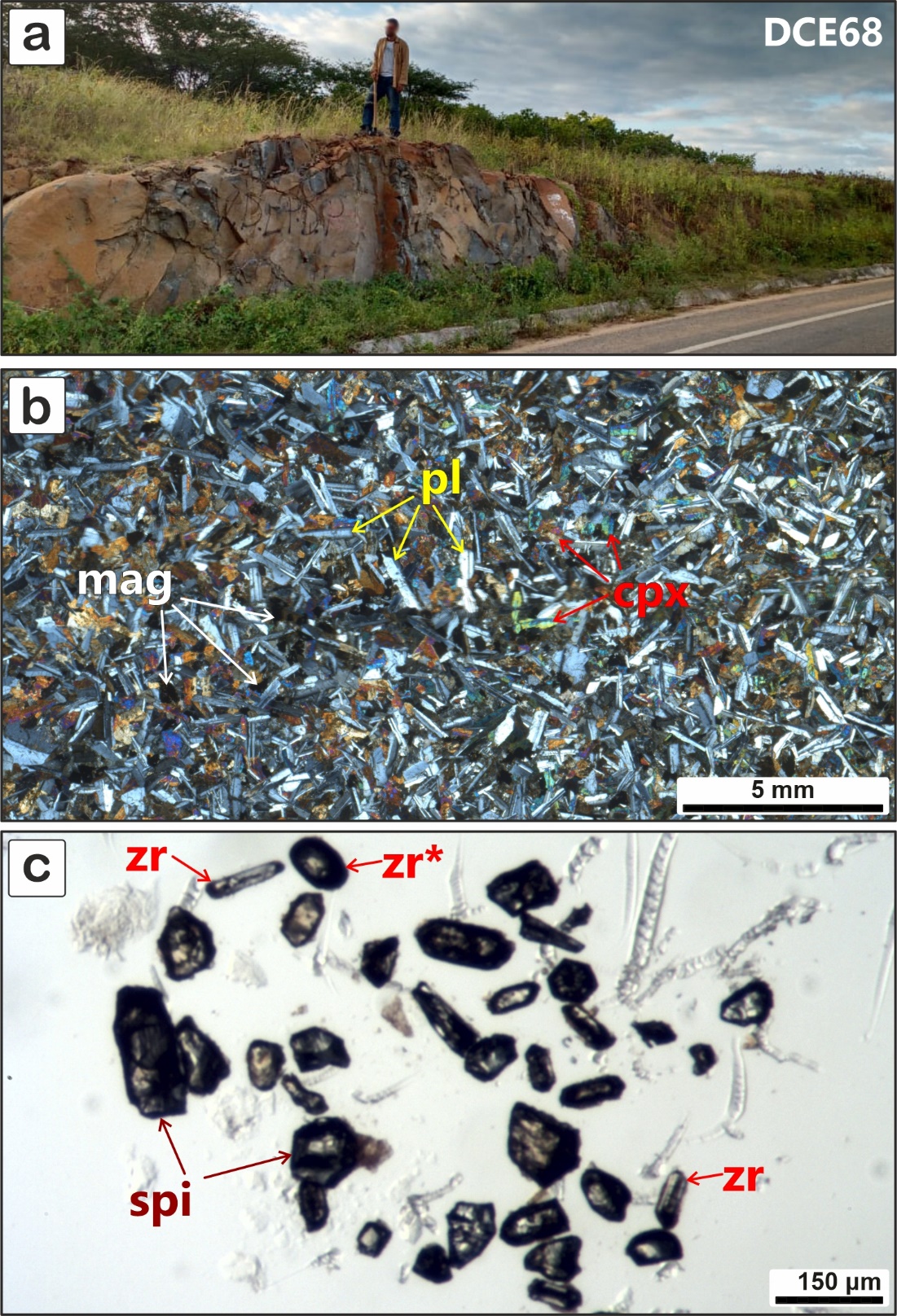 |
| --- |
| Figure S1 – A) Outcrop of DCE68 site alongside the CE-166 highway in the Ceará state, NE Brazil. B) Textural aspect of the Senador Pompeu dikes, holocrystalline/subophitic diabases composed mainly of plagioclase (pl), clinopyroxene (cpx) and magnetite (mag). C) Zircon (zr) crystals concentrated from sample DCE68; 7 crystals were handpicked for U-Pb dating. Spinels (spi) and inherited zircons (zr*) were also found. |

References Cited

1. Hollanda, M. H. B. M. *et al.* The Mesozoic Equatorial Atlantic Magmatic Province (EQUAMP): A New Large Igneous Province in South America. in *Dyke Swarms of the World: A Modern Perspective* (eds. Srivastava, R., Ernst, R. E. & Peng) 87–110 (Springer Singapore, 2019). doi:10.1007/978-981-13-1666-1.

2. Macêdo Filho, A. A. & Hollanda, M. H. B. M. Petrogenesis of Mesozoic giant dike swarms and geodynamical insights about Gough flavors near the Equatorial Atlantic margin (NE South America). *Lithos* **413**, 106611 (2022).

3. Merle, R. *et al.* 40Ar/39Ar ages and Sr-Nd-Pb-Os geochemistry of CAMP tholeiites from Western Maranhão basin (NE Brazil). *Lithos* **122**, 137–151 (2011).

4. Marzoli, A. *et al.* Extensive 200-million-year-old continental flood basalts of the Central Atlantic Magmatic Province. *Science (1979)* **284**, 616–618 (1999).

5. Mocitaiba, L. S. R., de Castro, D. L. & de Oliveira, D. C. Cartografia geofísica regional do magmatismo mesozoico na Bacia do Parnaíba. *Geologia USP - Serie Cientifica* **17**, 169–192 (2017).

6. de Castro, D. L., Oliveira, D. C. & Hollanda, M. H. B. M. Geostatistical Interplay Between Geophysical and Geochemical Data: Mapping Litho-Structural Assemblages of Mesozoic Igneous Activities in the Parnaíba Basin (NE Brazil). *Surv Geophys* **39**, 683–713 (2018).

7. Melo, A. C. C., de Castro, D. L., Fraser, S. J. & Macêdo Filho, A. A. Using self-organizing maps in airborne geophysical data for mapping mafic dyke swarms in NE Brazil. *J Appl Geophy* **192**, (2021).

8. Melo, A. C. C., de Castro, D. L., de Oliveira, D. C. & de Hollanda, M. H. B. M. Journal of South American Earth Sciences Mesozoic dike swarms in Borborema Province (NE Brazil): A structural analysis based on airborne geophysical data and field work. *J South Am Earth Sci* **113**, (2022).

9. Oliveira, A. L., Hollanda, M. H. B. M., Siqueira, R. & Macêdo Filho, A. A. Using a ‘speedy’ unspiked K-Ar methodology to investigate age patterns in giant mafic dike swarms. *Geological Society, London, Special Publications* **518**, 285–300 (2022).

10. Macêdo Filho, A. A. *et al.* Correlations among large igneous provinces related to the West Gondwana breakup: A geochemical database reappraisal of Early Cretaceous plumbing systems. *Geoscience Frontiers* **14**, 101479 (2023).

11. Oliveira, A. L., Pimentel, M. M., Fuck, R. A. & Oliveira, D. C. *Petrology of Jurassic and Cretaceous basaltic formations from the Parnaíba Basin, NE Brazil: Correlations and associations with large igneous provinces*. *Geological Society Special Publication* vol. 472 (Cratonic Basin Formation: A Case Study of the Parnaíba Basin of Brazil., 2018).

12. Heilbron, M. *et al.* Geochemical and temporal provinciality of the magmatism of the eastern Parnaíba Basin, NE Brazil. *Cratonic Basin Formation: A Case Study of the Parnaíba Basin of Brazil* **472**, 1–28 (2018).

13. de Min, A. *et al.* The Central Atlantic Magmatic Province (CAMP) in Brazil: Petrology, Geochemistry, 40Ar/39Ar Ages, Paleomagnetism and Geodynamic Implications. *Geophysical Monograph Series* **136**, 91–128 (2003).

14. Ernesto, M. *et al.* Paleomagnetic and geochemical constraints on the timing and duration of the CAMP activity in northeastern Brazil. *Geophysical Monograph Series* **136**, 129–149 (2003).

15. Fernandes, L. B. de M., de Sá, E. F. J., Vasconcelos, P. M. de P. & Córdoba, V. C. Structural controls and 40Ar/39Ar geochronological data of basic dike swarms in the eastern domain of the Parnaíba Basin, northeast Brazil. *J South Am Earth Sci* **101**, 102601 (2020).

16. Marzoli, A. *et al.* The Central Atlantic Magmatic Province (CAMP): A Review. in *The Late Triassic World* (ed. Tanner, L. H.) 91–125 (Topics in Geobiology 46, 2018). doi:10.1007/978-3-319-68009-5_4.
